# Bioinformatic and statistical analysis of the optic nerve head in a primate model of ocular hypertension

**DOI:** 10.1186/1471-2202-9-93

**Published:** 2008-09-26

**Authors:** Kenneth S Kompass, Olga A Agapova, Wenjun Li, Paul L Kaufman, Carol A Rasmussen, M Rosario Hernandez

**Affiliations:** 1 Department of Ophthalmology, Northwestern University, Chicago, IL 60611, USA; 2 Department of Ophthalmology and Visual Sciences, Washington University School of Medicine, St. Louis, MO 63110, USA; 3 Department of Ophthalmology and Visual Sciences, University of Wisconsin Medical School, Madison, WI 53792, USA

## Abstract

**Background:**

The nonhuman primate model of glaucomatous optic neuropathy most faithfully reproduces the human disease. We used high-density oligonucleotide arrays to investigate whole genome transcriptional changes occurring at the optic nerve head during primate experimental glaucoma.

**Results:**

Laser scarification of the trabecular meshwork of cynomolgus macaques produced elevated intraocular pressure that was monitored over time and led to varying degrees of damage in different samples. The macaques were examined clinically before enucleation and the myelinated optic nerves were processed post-mortem to determine the degree of neuronal loss. Global gene expression was examined in dissected optic nerve heads with Affymetrix GeneChip microarrays. We validated a subset of differentially expressed genes using qRT-PCR, immunohistochemistry, and immuno-enriched astrocytes from healthy and glaucomatous human donors. These genes have previously defined roles in axonal outgrowth, immune response, cell motility, neuroprotection, and extracellular matrix remodeling.

**Conclusion:**

Our findings show that glaucoma is associated with increased expression of genes that mediate axonal outgrowth, immune response, cell motility, neuroprotection, and ECM remodeling. These studies also reveal that, as glaucoma progresses, retinal ganglion cell axons may make a regenerative attempt to restore lost nerve cell contact.

## Background

The glaucomas are a multifactorial group of diseases with many different causes and one common endpoint: the loss of retinal ganglion cells of the retina, leading to thinning of the retinal nerve fiber layer and deficits in the visual field [1-3]. Ocular hypertension is the leading risk factor for glaucoma [4,5]. For human patients presenting with glaucoma, treatments that lower intraocular pressure are effective, even in cases where intraocular pressure is not abnormally elevated [6]. In animal models, interventions that produce elevated intraocular pressure lead to predictable retinal ganglion cell loss [7,8].

Astrocytes are the most abundant glial cells in the adult central nervous system. Normally, astrocytes provide metabolic and structural support to neurons and participate in the maintenance and detoxification of the extracellular space of the central nervous system. In neurodegenerative diseases or following central nervous system injury, quiescent astrocytes acquire a reactive phenotype and produce many enzymes, proteins, cytokines, and free radicals that are not produced under normal conditions [9,10].

As part of the change from quiescent astrocytes to reactive astrocytes, glaucomatous optic nerve head astrocytes exhibit differential expression of a large number of genes [11]. Reactive astrocytes in glaucomatous eyes may initially represent a cellular attempt to limit the extent of neuronal injury and to promote tissue repair, but reactive glial cells may also have noxious effects on optic nerve axons by creating mechanical injury and/or changing the microenvironment of neurons [12-14]. Previous studies from our laboratory reported that genes related to lipid synthesis and metabolism, steroid metabolism and glutathione metabolism were upregulated in optic nerve head astrocytes cultured from patients with primary open angle glaucoma [11,15,16]. In addition, reactive astrocytes in the glaucomatous optic nerve head engage in extracellular matrix (ECM) remodeling of the lamina cribrosa [12] leading to the cupping or excavation of the optic disc characteristic of glaucoma [17].

Our working model of damage in ocular hypertensive glaucoma states that abnormally elevated intraocular pressure converts normal optic nerve head astrocytes to "reactive" astrocytes, which are characterized by the increased expression of glial fibrillary acidic protein (GFAP) [10,18,19], a member of the intermediate filament family uniquely expressed by astrocytes and considered to be a hallmark of central nervous system injury [20]. There is evidence that elevated hydrostatic pressure can directly trigger astrocytes to assume the reactive phenotype [18,21,22].

Several recent studies have used whole genome microarrays to catalog changes in transcription that accompany glaucoma. These have included the analysis of primary cultures of human optic nerve head astrocytes from glaucomatous donors [11]; cultured human optic nerve head astrocytes from normal donors exposed to pressure for varying periods of time [23]; optic nerve head cells from a rat model of glaucoma [24]; and the retinal cells of several animal models of glaucoma, including the DBA/2J mouse [25], the cynomolgus macaque [26], and the rat [27]. There are substantial transcriptional differences between cells *in vivo *and *in vitro *[28-30]. Due to the unique anatomy and sophistication of the primate lamina cribrosa, nonhuman primate models are preferred for glaucoma research and replicate the disease with the highest fidelity [7,8]. This is because the nonhuman primate visual system, including the structure of the optic nerve head, is nearly identical to that of human [31,32]. This contrasts with mice, which do not have a lamina cribrosa, and rats, which have a very primitive lamina cribrosa [33,34]. The primate model develops visual field deficits and cupping that are indistinguishable from those in human glaucoma [1,35]. For the present study, we used a nonhuman primate model to investigate transcriptional changes in the cells of the optic nerve head during experimental glaucoma. High-density oligonucleotide microarrays identified global transcriptional changes in the nonhuman primate optic nerve head. We then compared these findings to the genes that are altered in immuno-enriched cultures of optic nerve head astrocytes from glaucomatous human donors.

## Results

### Elevation of intraocular pressure

**Figure 1:**
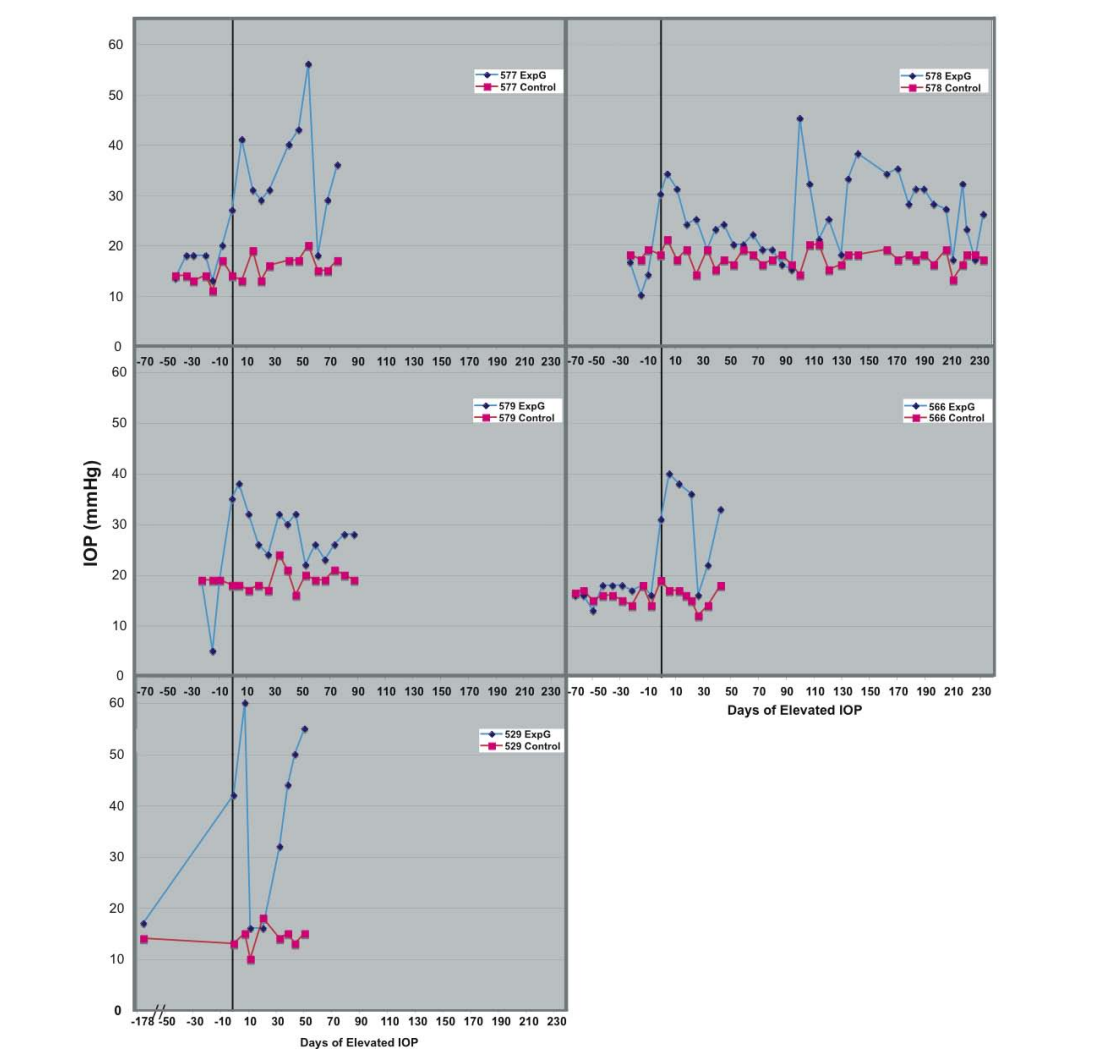
**Intraocular pressure measurements for five paired glaucomatous samples**. Intraocular pressure measurements from day of first laser in ExpG eye to sacrifice. Day 0 (vertical line) is the first time intraocular pressure was above 25 mm Hg.

Intraocular pressure readings from Goldmann applanation tonometry are shown in Figure 1 from the first reading above 25 mm Hg in each laser treated eye. Table 1 and Table 2 show physiological data for all samples processed for microarray.

**Table 1:** Control and ExpG cynomolgus macaques.

**Macaque**	**Age (years)**	**Sex**	**Axon loss (%)**	**Evaluation**	**Control/ExpG**
577	6	F	21	Mild	Paired
578	6	F	25	Mild	Paired
579	6	F	32	Mild	Paired
566	7	F	74	Advanced	Paired
529	5 1/2	M	59	Moderate	Paired
K605os	5	M	0	Control	Control
K605od	5	M	0	Control	Control
590os	4	F	0	Control	Control

All samples used for microarray and qRT-PCR in the present study. 'Axon loss' and 'Evaluation' are as defined in Methods. M = male; F = female.

**Table 2:** Clinical data for ExpG cynomolgus macaques.

**Macaque**	**IOP, control eye (mm Hg)**	**IOP, ExpG eye (mm Hg)**	**Duration elevated IOP (weeks/days)**	**C/D ratio control eye**	**C/D ratio ExpG eye**	**Axon loss (%)**	**Evaluation**
**577**	15.0 ± 2.6	24.7 ± 7.5	10.9/76	0.25	0.4	21	Mild
**578**	17.1 ± 2.4	33.2 ± 10.3	33.4/234	0.25	0.5	25	Mild
**579**	19.1 ± 1.8	28.1 ± 4.5	12.6/88	0.4	0.7	32	Mild
**566**	15.0 ± 2.7	27.4 ± 18.5	6.1/43	0.25	0.8	74	Advanced
**529**	13.8 ± 2.3	36.0 ± 18.5	7.3/51	0.2	0.9	59	Moderate

Experimental paired cynomolgus macaques used for the microarray analysis and qRT-PCR verification of the present study.

IOP measurements represent mean ± SD. 'Axon loss' and 'Evaluation' are as defined in Methods.

IOP = Intraocular pressure. C/D ratio = cup/disk ratio. M = male; F = female.

### Evaluation of damage

Fundus photographs are shown for samples 577 (21% axon loss), 578 (25% axon loss), and 566 (74% axon loss) [see Additional file 1]. These samples gave a representative picture of the progression of glaucomatous enlargement of the rim of the optic nerve head and thinning of the retinal nerve fiber layer.

Further *in vivo *evaluation of retinal nerve fiber layer (RNFL) thickness was made with the GDx VCC scanning laser polarimetry (SLP) with variable corneal compensation [see Additional file 2]. SLP is an ocular imaging technique based on the birefringence of the RNFL, and has been used to obtain reproducible quantitative measurements of the RNFL in human and monkey eyes [60]. The GDx VCC has a variable corneal polarization compensator (VCC) that allows for individual eye birefringence compensation, which increases the accuracy of the RNFL thickness assessment. GDxVCC images are color coded for thickness. Darker colors (e.g. blue) represent thinner areas of RNFL and bright colors (e.g. orange) represent thicker areas. Differences in SLP parameters between the experimental glaucoma (ExpG) eyes and healthy control eyes of the samples were assessed to quantify RNFL damage. Thinning in the typical glaucomatous, "hourglass" shape [61] was evident, with images showing more pronounced change from orange to blue coloration in the superior and inferior regions.

To further quantify disease progression, axon loss was measured from cross-sections of the optic nerve of each sample processed for microarray analysis (Table 2).

### Remapping of probe set definitions to Affymetrix human microarrays

Recent improvements in sequencing and genomic analysis have led to more accurate "definitions" that relate oligonucleotide sequences on the Affymetrix arrays to expressed RNAs [46]. We used these more accurate definitions (intended for human and chimpanzee) to update the probe sets of the Affymetrix HGU133A and HGU133Av2 oligonucleotide microarrays used in this study. Limited genomic information is available for the cynomolgus macaque, the species used for this study. However, the close evolutionary relationship of humans and chimpanzees to the macaque permits microarray analysis using human chips and gene definitions from either humans or chimpanzees. Because the cynomolgus macaque is evolutionarily approximately equidistant from humans and chimpanzees, there is theoretically no advantage in using either the human or the chimpanzee definitions for the analysis of transcripts from the macaque. We performed the microarray analysis in duplicate, using definitions for both human and chimpanzee. The Affymetrix detection call algorithm reliably eliminates probe sets that, due to sequence variations between species, do not hybridize accurately [56]. It was used to eliminate false hybridization signals (see Methods). As our most compelling targets were verified using qRT-PCR, any candidates generated with either of the two sets of definitions could be verified with this more precise technique. Using human UniGene-based definitions, 4,357 probesets were detected in 16/33 arrays, and 4,358 probesets were detected in 14/28 arrays (for all five ExpG eyes compared to eight control eyes); using chimpanzee UniGene-based definitions, 3,844 probesets were detected in 16/33 arrays, and 3,842 probesets were detected in 14/28 arrays (for three mild ExpG eyes compared to eight control eyes).

### Results of statistical testing

When comparing microarray data from the five ExpG eyes with the control group of eight eyes, the UniGene-based chimpanzee definitions identified 30 significant, differentially expressed probe sets, and the UniGene-based human definitions produced 33 significant, differentially expressed probe sets (Table 3). Nineteen significant, differentially expressed probe sets were shared between the two groups. Human definitions identified guanine nucleotide-binding protein, gamma-transducing activity polypeptide 1 (GNGT1), which was likely introduced as a retina-specific contaminant during the dissection [62]. Chimpanzee definitions identified phosducin (PDC), also likely a retina-specific contaminant [63]. For this reason, GNGT1 and PDC were removed from further consideration. Fold change (i.e. fold increase or fold decrease) and standard error of fold change were nearly identical for transcripts identified using both definitions.

**Table 3:** Significant differentially expressed probesets from five ExpG eyes compared to eight control eyes by microarray.

**PROBESET**	**Definition**	**Group fold change**	**Representative public ID**	**Gene title**	**Gene symbol**	**RP**	**SAM**	**ONH astrocyte**	**RGC**
Hs.103291	HS, PT	1.79 ± 0.68	NM_016588	neuritin 1	NRN1	0.00	0.01	NA	Y
Hs.12333	HS, PT	-1.69 ± 0.25	NM_007282	ring finger protein 13	RNF13	0.03	0.01	Y	NA
Hs.134974	HS, PT	1.70 ± 0.37	NM_002045	growth associated protein 43	GAP43	0.06	0.01	N	Y
Hs.155097	HS, PT	-1.62 ± 0.49	M36532	carbonic anhydrase II	CA2	0.05	0.11	N	NA
Hs.155247	HS	-2.37 ± 0.94	NM_005165	aldolase C, fructose-bisphosphate	ALDOC	0.00	0.01	Y	Y
Hs.165636	HS, PT	1.88 ± 0.54	NM_017594	DIRAS family, GTP-binding RAS-like 2	DIRAS2	0.00	0.00	NA	NA
Hs.198760	HS	8.37 ± 4.66	X15306	neurofilament, heavy polypeptide 200 kDa	NEFH	0.00	0.00	N	Y
Hs.233240	HS, PT	1.66 ± 0.60	NM_004369	collagen, type VI, alpha 3	COL6A3	0.03	0.11	Y	NA
Hs.235873	HS, PT	-1.61 ± 0.48	NM_024897	progestin and adipoQ receptor family member VI	PAQR6	0.01	0.08	NA	NA
Hs.277035	HS	1.48 ± 0.20	BC006230	monoglyceride lipase	MGLL	0.31	0.01	Y	NA
Hs.308709	HS	-2.22 ± 0.88	NM_005313	protein disulfide isomerase, family A, member 3	PDIA3	0.03	0.15	Y	NA
Hs.351279	HS, PT	1.90 ± 0.56	X76775	major histocompatibility complex, class II, DM alpha	HLA-DMA	0.02	0.01	N	NA
Hs.369397	HS, PT	1.63 ± 0.32	NM_000358	transforming growth factor, beta-induced, 68 kDa	TGFBI	0.05	0.00	Y	NA
Hs.378901	HS, PT	-1.49 ± 0.30	NM_014717	zinc finger protein 536	ZNF536	0.06	0.03	N	NA
Hs.412355	HS	1.82 ± 0.83	NM_001492	growth differentiation factor 1/LAG1 homolog, ceramide synthase 1	GDF1/LASS1	0.01	0.08	Y	NA
Hs.435557	HS, PT	-1.75 ± 0.39	BF059313	kinesin family member 5C	KIF5C	0.02	0.00	N	NA
Hs.443625	HS, PT	2.01 ± 0.63	NM_000090	collagen, type III, alpha 1 (Ehlers-Danlos syndrome type IV, autosomal dominant)	COL3A1	0.01	0.01	Y	NA
Hs.458573	HS, PT	1.47 ± 0.20	NM_006207	platelet-derived growth factor receptor-like	PDGFRL	0.15	0.00	Y	NA
Hs.478153	HS, PT	-1.62 ± 0.45	NM_005025	serpin peptidase inhibitor, clade I (neuroserpin), member 1	SERPINI1	0.03	0.14	Y	Y
Hs.483444	HS	1.60 ± 0.49	AF144103	chemokine (C-X-C motif) ligand 14	CXCL14	0.03	0.07	NA	NA
Hs.485130	HS	1.79 ± 0.52	NM_002121	major histocompatibility complex, class II, DP beta 1	HLA-DPB1	0.03	0.02	Y	NA
Hs.489142	HS, PT	1.62 ± 0.42	NM_000089	collagen, type I, alpha 2	COL1A2	0.03	0.01	Y	NA
Hs.48924	HS	-1.85 ± 0.46	NM_014782	armadillo repeat containing, X-linked 2	ARMCX2	0.03	0.03	Y	NA
Hs.496622	HS, PT	-1.80 ± 0.41	NM_005032	plastin 3 (T isoform)	PLS3	0.03	0.03	Y	NA
Hs.515354	HS	-1.73 ± 0.58	NM_002361	myelin-associated glycoprotein	MAG	0.05	0.23	Y	NA
Hs.515369	HS	1.77 ± 0.55	NM_003332	TYRO protein tyrosine kinase binding protein	TYROBP	0.03	0.14	N	NA
Hs.518525	HS	-1.60 ± 0.30	NM_002065	glutamate-ammonia ligase (glutamine synthetase)	GLUL	0.07	0.00	Y	NA
Hs.520048	HS	1.50 ± 0.56	M60333	major histocompatibility complex, class II, DR alpha	HLA-DRA	0.03	0.51	Y	NA
Hs.529571	HS, PT	1.63 ± 0.44	NM_002899	retinol binding protein 1, cellular	RBP1	0.02	0.03	Y	Y
Hs.550276	HS, PT	1.59 ± 0.23	NA	NA	NA	0.07	0.00	Y	NA
Hs.70327	HS	1.68 ± 0.38	NM_001311	cysteine-rich protein 1 (intestinal)	CRIP1	0.03	0.01	Y	NA
Hs.88778	HS, PT	-1.90 ± 0.44	BC002511	carbonyl reductase 1	CBR1	0.03	0.01	Y	NA
Hs.109225	PT	1.58 ± 1.42	NM_001078	vascular cell adhesion molecule 1	VCAM1	0.04	0.48	Y	NA
Hs.144197	PT	-1.74 ± 0.50	NM_003360	UDP glycosyltransferase 8 (UDP-galactose ceramide galactosyltransferase)	UGT8	0.02	0.06	N	NA
Hs.190495	PT	6.78 ± 4.64	NM_002510	glycoprotein (transmembrane) nmb	GPNMB	0.00	0.01	Y	Y
Hs.388664	PT	1.53 ± 0.50	NM_000975	ribosomal protein L11	RPL11	0.03	0.47	Y	NA
Hs.502235	PT	2.30 ± 1.03	NA	NA	NA	0.01	0.02	Y	Y
Hs.518773	PT	-2.22 ± 0.58	BF448062	ubiquitin-conjugating enzyme E2D 3 (UBC4/5 homolog, yeast)	UBE2D3	0.04	0.01	Y	NA
Hs.530902	PT	1.63 ± 0.42	NM_005849	immunoglobulin superfamily, member 6	IGSF6	0.10	0.02	N	NA
Hs.532768	PT	1.60 ± 0.39	NM_002615	pigment epithelium derived factor/serpin peptidase inhibitor, clade F, member 1	PEDF/SERPINF1	0.13	0.02	Y	NA
Hs.58414	PT	1.69 ± 0.63	NM_001458	filamin C, gamma (actin binding protein 280)	FLNC	0.04	0.09	Y	NA
Hs.75285	PT	1.49 ± 0.39	NM_002216	inter-alpha (globulin) inhibitor H2	ITIH2	0.05	0.03	N	NA

'Definition' indicates the species definitions used; 'HS' indicates human; 'PT' indicates chimpanzee. For transcripts identified by both definitions (e.g. NRN1), the data shown in the table was produced using the human definition. Fold change (i.e. fold increase or fold decrease) and standard error of fold change were calculated as described in Methods. 'RP' indicates p-values generated by Rank Products; 'SAM' indicates p-values generated by significance analysis of microarrays. 'ONH Astrocyte' and 'RGC' indicate probable expression in optic nerve head astrocytes and retinal ganglion cells, respectively (Methods); 'NA' indicates no data were available.

The same analysis was performed after omitting the two most advanced glaucoma samples (529, 566) to identify differentially expressed probe sets that were preferentially expressed during the mild stages of experimental glaucoma (Table 4).

**Table 4:** Significant differentially expressed probesets from three mild ExpG eyes compared to eight control eyes by microarray.

**PROBESET**	**Definition**	**Group Fold Change**	**Representative Public ID**	**Gene Title**	**Gene Symbol**	**RP**	**SAM**	**ONH Astrocyte**	**RGC**
Hs.103291	HS, PT	2.19 ± 0.87	NM_016588	neuritin 1	NRN1	0.03	0.07	NA	Y
Hs.165636	HS, PT	2.21 ± 0.65	NM_017594	DIRAS family, GTP-binding RAS-like 2	DIRAS2	0.02	0.01	NA	NA
Hs.198760	HS, PT	4.67 ± 2.47	X15306	neurofilament, heavy polypeptide 200 kDa	NEFH	0.02	0.20	N	Y
Hs.369397	HS, PT	1.86 ± 0.46	NM_000358	transforming growth factor, beta-induced, 68 kDa	TGFBI	0.11	0.05	Y	NA
Hs.515369	HS	1.63 ± 0.23	NM_003332	TYRO protein tyrosine kinase binding protein	TYROBP	0.20	0.01	N	NA
Hs.529571	HS, PT	1.94 ± 0.48	NM_002899	retinol binding protein 1, cellular	RBP1	0.08	0.02	Y	Y
Hs.75318	HS, PT	1.57 ± 0.20	AL565074	tubulin, alpha 4a	TUBA4A	0.20	0.02	Y	NA
Hs.211914	PT	1.65 ± 0.22	BC005954	NADH dehydrogenase (ubiquinone) Fe-S protein 7, 20 kDa (NADH-coenzyme Q reductase)	NDUFS7	0.23	0.01	Y	NA
Hs.512610	PT	1.76 ± 0.36	NM_016199	LSM7 homolog, U6 small nuclear RNA associated (S. cerevisiae)	LSM7	0.15	0.02	Y	NA

This comparison was performed to preferentially identify transcripts that changed during the mild stages of glaucomatous axonal damage. Samples 577, 578, and 579 had axonal losses ranging from 25%–32% (**Table 2**) and were tested separately. Results are shown here. Samples 566 and 529 had more severe axonal loss (74% and 59%, respectively; **Table 2**) and were excluded from this comparison. 'Definition' indicates the species definitions used; 'HS' indicates human; 'PT' indicates chimpanzee. For transcripts identified by both definitions (e.g. NRN1), the data shown in the table were produced using the human definition. Fold change (i.e. fold increase or fold decrease) and standard error of fold change were calculated as described in Methods. 'RP' indicates indicates p-values generated by Rank Products; 'SAM' indicates indicates p-values generated by significance analysis of microarrays. 'ONH Astrocyte' and 'RGC' indicate probable expression in optic nerve head astrocytes and retinal ganglion cells, respectively (see Methods); 'NA' indicates no data were available.

### Prediction of cell types expressing differentially expressed genes

We were most interested in genes differentially expressed by optic nerve head astrocytes and retinal ganglion cells during experimental glaucoma. To estimate which genes were expressed by optic nerve head astrocytes, detection call information from previously published Affymetrix datasets from this laboratory was used [11,23]. Retinal ganglion cell expression data from one EST and one microarray study was obtained, respectively, from [58] and [59]. Both microarray tables include columns with optic nerve head astrocyte and retinal ganglion cell expression data for the significant transcripts (Table 3, Table 4).

### Validation of selected differentially expressed genes by real-time RT-PCR

Transcripts selected from Table 3 (GAP43, GPNMB, NEFH), Table 4 (NEFH), or the results of individual paired comparisons (APOE, BMP2, CAPG, NEFL, STMN2) [see Additional file 3] were confirmed by quantitative real time PCR in the two most mild (577, 578) and two most advanced (566, 529) experimental, paired samples. Results are shown in Table 5 as relative gene expression values (fold change) between the laser-treated eye and the normal contralateral eye. There was good correlation between the quantitative real time PCR data and the microarray results. Primer sequences are provided [see Additional file 4].

**Table 5:** qRT-PCR verification of selected transcripts in ExpG.

**Transcript**		**577 FX**	**578 FX**	**566 FX**	**529 FX**
**APOE**	**qRT-PCR**	**1.11 ± 0.13**	**1.15 ± 0.16**	**3.55 ± 0.38**	**3.07 ± 0.38**
	Microarray	1.75 ± 0.24	-1.15 ± 0.15	2.95 ± 0.52	1.24 ± 0.16
**BMP2**	**qRT-PCR**	**1.92 ± 0.13**	**-1.29 ± 0.19**	**3.30 ± 0.16**	**3.25 ± 0.35**
	Microarray	1.34 ± 0.06	-1.30 ± 0.12	2.19 ± 0.36	2.14 ± 0.56
**CAPG**	**qRT-PCR**	**1.26 ± 0.09**	**1.72 ± 0.25**	**5.70 ± 0.35**	**1.66 ± 0.16**
	Microarray	1.54 ± 0.20	1.05 ± 0.09	3.93 ± 0.36	-1.13 ± 0.15
**GAP43**	**qRT-PCR**	**2.49 ± 0.15**	**1.39 ± 0.24**	**3.32 ± 0.33**	**3.18 ± 0.50**
	Microarray	1.52 ± 0.07	-1.04 ± 0.04	2.83 ± 0.26	1.55 ± 0.21
**GPNMB**	**qRT-PCR**	**2.40 ± 0.15**	**3.17 ± 0.31**	**90.68 ± 9.07**	**3.77 ± 0.27**
	Microarray	1.68 ± 0.26	1.27 ± 0.28	29.64 ± 6.44	2.04 ± 0.50
**NEFH**	**qRT-PCR**	**1.74 ± 0.24**	**4.86 ± 0.35**	**1.65 ± 0.14**	**56.48 ± 6.53**
	Microarray	2.95 ± 0.21	6.18 ± 0.85	2.53 ± 0.26	42.68 ± 4.88
**NEFL**	**qRT-PCR**	**2.46 ± 0.14**	**7.75 ± 0.37**	**-1.63 ± 0.13**	**5.38 ± 0.97**
	Microarray	2.22 ± 0.14	3.59 ± 0.21	1.15 ± 0.06	1.71 ± 0.29
**STMN2**	**qRT-PCR**	**2.44 ± 0.10**	**13.12 ± 0.95**	**-1.04 ± 0.07**	**4.03 ± 0.33**
	Microarray	1.69 ± 0.11	3.46 ± 0.32	-1.04 ± 0.06	1.77 ± 0.36

qRT-PCR verification in four paired glaucomatous macaques of eight selected transcripts. Fold change (i.e. fold increase or fold decrease; 'FX') was calculated within each paired sample as described in Methods. Standard error of fold change was calculated as described in Methods.

Numerical values for microarray are from human definitions, with the exception of GPNMB, which was not detected by human definitions.

### Histological confirmation of selected genes

To further investigate genes identified by microarray, laser treated cynomolgus and rhesus macaque samples with mild to moderate axon loss [see Additional file 5 and Additional file 6] were processed for immunohistochemistry with antibodies to growth-associated protein 43 (GAP43), glycoprotein transmembrane non-metastatic B (GPNMB), and neurofilament heavy (NEFH). GPNMB was chosen because it is a null mutation in the DBA/2J mouse strain, which develops iris pigment dispersion syndrome and iris stromal atrophy; the former was mapped to a premature stop mutation in GPNMB [64,65]; NEFH and GAP43 were chosen because they are markers of axonal regrowth [66,67], which has not been previously described in a primate glaucoma model.

**Figure 2:**
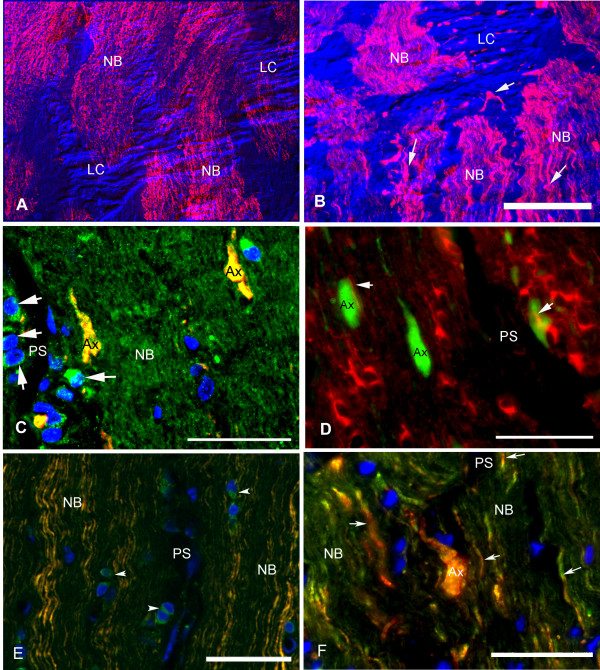
**Axonal morphology of monkey optic nerves with experimental glaucoma in sagittal sections of the ONH (Macaque #2; Supplemental Table 3)**. (**A**) Sagittal view of the normal optic nerve head stained for neurofilament heavy (red, NF200). Superimposed in blue is the DIC image of the structure of the lamina cribrosa (LC). (**B**) Similar view of the contralateral ExpG eye. Note irregular size and morphology of the axons (arrows) in the optic nerve head in experimental glaucoma (A and B, scale bar, 50 μm). NB = nerve bundles. (**C**) Co-localization of GAP43 (green) and phosphorylated NEFH (red) in bulb-like structures (Ax) in ExpG. Astrocytes (arrows) in the pial septa (PS) show abundant GAP43 staining. NB = nerve bundle (scale bar, 25 μm). (**D**) Double immunostaining for stathmin (red) and phosphorylated neurofilament (green) shows oligodendrocyte processes (arrows) on axonal bulb-like structures (Ax) in ExpG. PS = pial septa (scale bar, 25 μm). (**E**) Co-localization of protein disulfide isomerase (PDI), a marker of endoplasmic reticulum, and phosphorylated NEFH (SMI31) in some axons in the nerve bundles (NB) in normal post laminar optic nerve suggested the presence of ribosomes, seen as green granules within axons. Notice that glial cells in the pial septa (PS) (arrowheads) stain positive for PDI (scale bar, 40 μm) (**F**). PDI and phosphorylated NEFH co-localization in axons in nerve bundles (NB) in ExpG (arrows). Abnormal axon (Ax) stained with NEFH and PDI in ExpG optic nerve (scale bar, 25 μm). Nuclei are stained with DAPI.

Two NEFH antibodies were used to examine the localization of upregulated NEFH in the axons of optic nerves of healthy and experimental glaucoma monkeys. NEFH immunohistochemistry with an antibody that recognizes phosphorylated and non-phosphorylated NEFH shows axons in the normal sample traversing through the lamina cribrosa in well-organized bundles (Figure 2A), while the axons in ExpG were disorganized with an abnormal axonal morphology (Figure 2B). Staining with an antibody that recognizes predominantly phosphorylated NEFH revealed presumptive growth cone-like structures in the post laminar optic nerves of monkeys with experimental glaucoma, which also were positive for GAP43 (Figure 2C), whereas these structures were absent in control monkeys. Optic nerve astrocytes expressed abundant GAP-43 in ExpG. Stathmin+ oligodendrocyte processes encircled the axonal bulbs in ExpG (Figure 2D). The growth cone-like structures were observed in all ExpG optic nerves examined. Double staining with NEFH and protein disulfide isomerase (PDI), a marker of endoplasmic reticulum vesicles, suggested new protein synthesis in axons [68] and glial cells in the optic nerve (Figure 2E, F).

**Figure 3:**
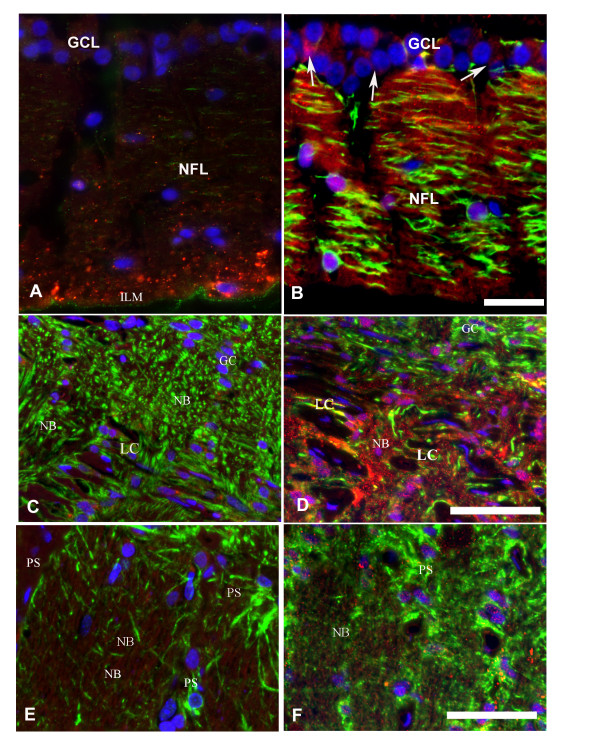
**Expression of GPNMB in experimental glaucoma in sagittal sections of the ONH (Macaque #3; Supplemental Table 3)**. (**A**) Low expression of GPNMB (red) in the retinal ganglion cell layer (GCL) and nerve fiber layer (NFL) in control monkey eye. Granular staining of GPNMB is present in the area adjacent to the inner limiting membrane (ILM). Very low staining for GFAP (green) is observed in the normal nerve fiber layer. Nuclei are stained with DAPI. (**B**) Upregulation of GPNMB in retinal ganglion cells (GCL) and in axons and retinal astrocytes in the nerve fiber layer in ExpG. Notice marked increase in GFAP staining in the nerve fiber layer. A, B: Magnification bar = 25 μm. (**C**) Low expression of GPMNB in astrocytes and axons in the normal monkey optic nerve head. (**D**) Marked upregulation of GPNMB in axons in the nerve bundles (NB) and in GFAP+ astrocytes (green) in the lamina cribrosa (LC) and glial columns (GC). Note that GPNMB appears to localize to the nucleus and cytoplasm in astrocytes in ExpG. C, D: Magnification bar = 40 μm. (**E and F**) Low expression of GPNMB associated with astrocytes and axons in the post laminar optic nerve. (E, Control; F, ExpG). E, F: Magnification bar = 40 μm.

GPNMB immunohistochemistry is shown in Figure 3. In control samples, GPNMB protein was detected in the retinal ganglion cell and nerve fiber layers of the retina (Figure 3A). In ExpG samples, GPNMB protein was sharply upregulated in retinal ganglion cells and their axons, and in retinal astrocytes in the retinal nerve fiber layer (Figure 3B). Within the optic nerve head, healthy samples had baseline expression levels of GPNMB protein in astrocytes and retinal ganglion cell axons (Figure 3C). In ExpG samples, GPNMB was sharply upregulated in retinal ganglion cells and their axons, as well as optic nerve head astrocytes (Figure 3D). GPNMB staining appeared to localize to the membranes of GFAP+ astrocytes. In contrast, GFAP+ astrocytes in the lamina cribrosa exhibited strong GPNMB staining where, at higher magnification, GPNMB protein could be seen around and over the nucleus in ExpG. Abundant GPNMB was also present in axons in the nerve bundles at the level of the lamina cribrosa (Figure 3D). Low levels of GPNMB were present in astrocytes, oligodendrocytes and nerve fibers in the myelinated post laminar optic nerve in normal and in ExpG (Figure 3E, F)

**Figure 4:**
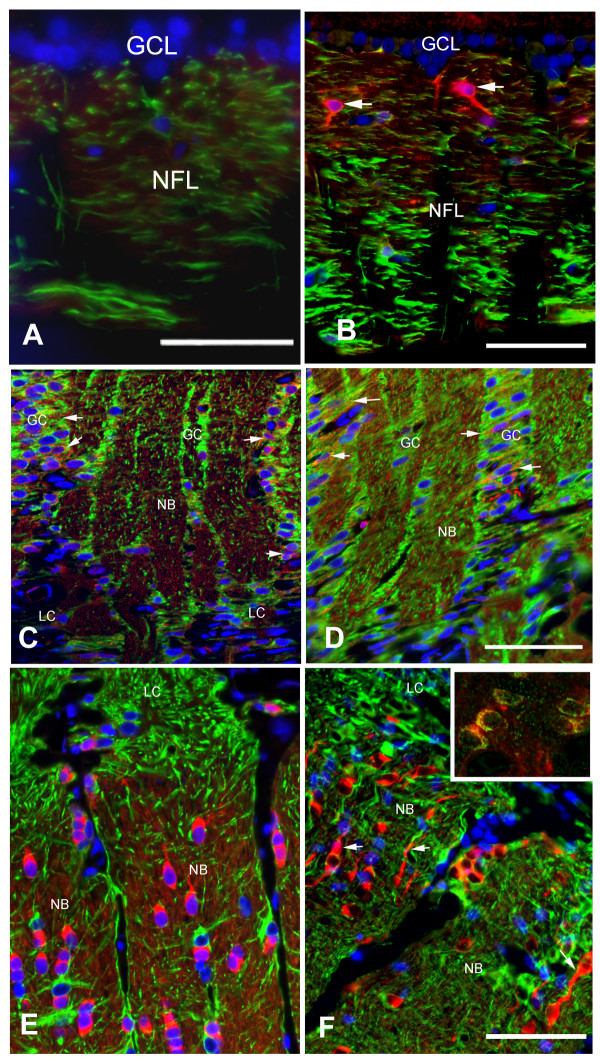
**Stathmin protein expression in experimental glaucoma in sagittal sections of the ONH (Macaque #5; Supplemental Table 3)**. (**A**) Stathmin (red) is not expressed in the retinal ganglion cell layer (GCL) or in the retinal nerve fiber layer (NFL) in the retina of normal monkeys. GFAP stains retinal astrocytes (green). (**B**) In ExpG, stathmin staining is present in the nerve fiber layer presumably in axons. Stathmin does not co-localize with GFAP+ retinal astrocytes in the nerve fiber layer. Some cells bearing processes stain strongly for stathmin in the nerve fiber layer (arrows). Magnification bar: A = 25 μm; B = 40 μm. (**C, D**) Co-localization of stathmin and GFAP in astrocytes of the glial columns (GC) in the optic nerve head in normal optic nerve head and ExpG. Axons in the nerve bundles (NB) also exhibit staining for stathmin. Note that cells in the lamina cribrosa (LC) are devoid of staining for stathmin. Magnification bar = 55 μm. (**E**) Stathmin stains oligodendrocytes in the post-laminar optic nerve in normal optic nerves. (**F**) Stathmin-labeled bipolar oligodendrocytes (arrows) are abundant in the post-laminar optic nerve in ExpG. Insert shows co-localization of APC (green), a marker of oligodendrocytes cell bodies and non-myelinating processes with stathmin in the post laminar optic nerve. Magnification bar = 35 μm.

Labeling with stathmin (STMN) antibody revealed specific upregulation of STMN in ExpG. Within the retina, STMN immunoreactivity was very low in normal eyes (Figure 4A). In ExpG, STMN was detected in the nerve fiber layer and in few GFAP-negative process-bearing cells. Retinal ganglion cells and retinal astrocytes did not exhibit STMN immunoreactivity (Figure 4B). In the prelaminar optic nerve head, low levels of STMN were expressed in the glial columns and in the nerve bundles in both control and ExpG samples without obvious differences (Figure 4C, D). In both control and ExpG, STMN expression was absent from the lamina cribrosa and present in the optic nerve proper. Oligodendrocytes exhibited strong immunoreactivity for STMN in the somata and cell processes (Figure 4E, F). In ExpG, many oligodendrocytes exhibited bipolar shape with long processes (Figure 4F). In control eyes STMN stained the nerve bundles, but not in ExpG (Figure 4E, F). Positive staining with adenomatous polyposis coli (APC) identified these cells as oligodendrocytes (inset, Figure 4F). STMN was not expressed in GFAP+ astrocytes in the myelinated nerve.

These results were consistent amongst all the samples, as were the negative controls for specificity of the primary and secondary antibodies. Staining in cynomolgus ONH was not different from the rhesus ONH samples.

### Confirmation of the expression of selected genes in human optic nerve head astrocytes

**Figure 5:**
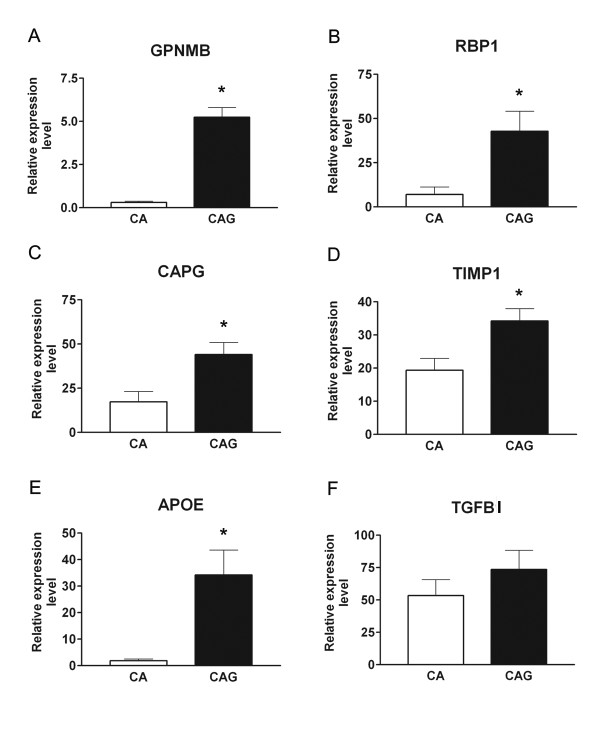
**Real time quantitative RT-PCR from human donor ONH astrocytes**. qRT-PCR was performed using mRNA isolated from primary cultures of ONH astrocytes derived from nine donors with primary open angle glaucoma (CAG) and nine age-matched Caucasian American normal donors (CA; **Supplemental Table 2**). Clear bars indicate control samples (CA) and filled-in bars indicate glaucomatous samples (CAG). Values represent the mean ± SE of relative expression normalized to 18S of each group of normal and glaucomatous astrocytes. For each donor, reactions were carried out in triplicate. **A**. GPNMB, fold change = 16.81; p = 0.0004. **B**. RBP1, fold change = 6.02; p = 0.02. **C**. CAPG fold change = 2.55; p = 0.02. **D**. TIMP1, fold change = 1.77; p = 0.02. **E**. APOE, fold change = 17.91; p = 0.04. **F**. TGFBI, fold change = 1.58; p > 0.05.

qRT-PCR was used to examine the expression of GPNMB, RBP1, CAPG, APOE, TGFBI, and TIMP1, which were identified in the nonhuman primate model, in immuno-enriched optic nerve head astrocytes from glaucomatous Caucasian American donors [see Additional file 7 and Additional file 8]. Figure 5 shows qRT-PCR confirmation of differential expression of the five genes in immuno-enriched optic nerve head astrocytes from healthy and glaucomatous human donors. CAPG, GPNMB, RBP1, APOE and TIMP1 were significantly upregulated in astrocytes from glaucomatous donors, consistent with their upregulation in the tissues of the macaque optic nerve head with ExpG. TGFBI was upregulated significantly by microarray (Table 3, Table 4), but not by qRT-PCR (Fig 5F). Two genes, NEFH and GPNMB, exhibited higher fold increases in samples with moderate to advanced glaucoma. In sample 566, GPNMB variation may be due to infiltration of the ONH with microglia or macrophages, as has been previously reported in advanced glaucoma in humans [69]. The higher NEFH increase in sample 529 may be due to retinal contamination or to higher IOP exposure in that spontaneous glaucoma compared to the laser treated samples.

## Discussion

We used the nonhuman primate model of glaucoma to investigate transcriptional changes occurring at the optic nerve head, the region thought to be the primary site of damage to retinal ganglion cells in glaucoma [70-73]. We validated selected transcripts with qRT-PCR, which generally agreed with microarray in direction and, to a lesser degree, magnitude. Our data was consistent with others' observations that larger fold changes in Affymetrix chips, with updated definitions [46], correlated best with qRT-PCR [55]. In addition, we used human optic nerve head astrocytes from normal and glaucomatous human donors to demonstrate independent upregulation of five putative astrocyte genes selected from the microarray data in monkeys with experimental glaucoma.

Previous studies have shown that updated sequence definitions for Affymetrix GeneChips produced improved estimates of differential expression [46,74-78]. In the present study, updated definitions produced more 'present' transcripts by the detection call algorithm [56,57] (data not shown), indicating that more hybridized transcripts were accurately detected.

Significant differentially expressed transcripts could be divided into five categories, and some have putative or established roles in multiple categories. Axonal elongation and synaptic plasticity genes, including growth-associated protein 43 (GAP43), neurofilament heavy (NEFH), and neuritin 1 (NRN1), preferentially expressed by retinal ganglion cells, indicated an attempt to re-extend lost or damaged axons. There was an upregulation of neuroprotective genes, including apolipoprotein E (APOE), cellular retinol binding protein 1 (RBP1), and pigment epithelium derived factor (PEDF/SERPINF1). We confirmed the upregulation of RBP1 in immuno-enriched optic nerve head astrocytes from glaucomatous human donors. This suggested that optic nerve head astrocytes attempted to facilitate retinal ganglion cell axon outgrowth by upregulation of neuroprotective molecules. Differential expression of motility genes, including capping protein G (CAPG) and transforming growth factor beta-induced (TGFBI), was consistent with the reactive, migratory astrocyte phenotype characteristic of glaucoma [12], as were ECM remodeling genes, such as GPNMB [79,80] and TIMP1 [81,82]. Immune genes, such as TYRO protein tyrosine kinase binding protein (TYROBP), major histocompatibility complex class II DM alpha (HLA-DMA), major histocompatibility complex class II DP beta 1 (HLA-DPB1), and major histocompatibility complex class II DR alpha (HLA-DRA), were likely expressed by infiltrating mononuclear cells, resident microglia, macrophages, and possibly also astrocytes acting as non-professional antigen-presenting cells [83-86]. The most compelling candidates for investigation are discussed further below in the context of glaucoma.

The appearance of GAP43+ and phospho-NEFH+ growth cone-like structures in the optic nerve has not been previously reported in the nonhuman primate model or in human glaucoma. It is thought that primate retinal ganglion cells do not regenerate damaged axons *in vivo*, including in glaucoma [87]. However, axon regeneration, accompanied by new protein synthesis in axons, was reported in dorsal root ganglia [88]. Our finding suggests that there may be plasticity in the adult primate optic nerve and possibly a window period, early in hypertensive glaucoma, when retinal ganglion cell axons attempt to regenerate. If true, this would underscore the importance of early diagnosis and early treatment of glaucoma, as post-mortem histological studies have shown that up to half of retinal ganglion cells are typically lost in glaucoma before any visual field defects are present. Alternatively, these structures may represent retraction bulbs that are characteristic of the disorganized microtubule network of damaged axons [89]. Future experiments will distinguish between these possibilities.

Capping protein G (CAPG) is a member of the gelsolin-villin family, and modulates cellular motility by capping actin filaments [90]. The importance of astrocyte motility in the progression of glaucoma is a present focus of investigation [24]; the upregulation of CAPG in the experimental glaucoma samples as well as in immuno-enriched optic nerve head astrocytes from Caucasian American donors with glaucoma makes it a strong candidate as an astrocyte migratory gene in glaucoma. Further, increased expression of capping protein G (CAPG) was shown previously by microarray and Western blot in purified human optic nerve head astrocytes from glaucomatous donors [11]. There is evidence that CAPG is regulated by elements of the AP-1 transcription factor complex [91], which was activated in the primate model of glaucoma [18]. CAPG has been shown to promote matrix metalloproteinase (MMP) activity [92]. The MMPs are a family of powerful ECM degrading enzymes [93] that may facilitate optic nerve head excavation in glaucoma [81,82,94].

GPNMB is a 560 amino acid, type I transmembrane glycoprotein. In the present study, GPNMB was sharply upregulated in optic nerve head astrocytes of Caucasian American donors with glaucoma. Roles have been ascribed to GPNMB in the immune system [95,96], in stress responses [97], and in ECM remodeling [79,80]. The latter has been underscored by roles for GPNMB in tumor invasion [98,99], where GPNMB promoted invasiveness through MMPs and also induced activation of epidermal growth factor receptor (EGFR) [80]. EGFR activation is characteristic of reactive astrocytes [9,100] and has been shown in glaucoma [13,101].

The DBA/2J mouse develops pigmentary glaucoma from the release of pigment and degenerated cellular components of the iris pigment epithelium and stroma that alter drainage structures, elevating intraocular pressure [102]. Two distinct eye diseases produce glaucoma in the DBA/2J strain – iris pigment dispersion syndrome, which was mapped to a null mutation in GPNMB, and iris stromal atrophy, which was mapped to a mutation in TYRP1 [64,65]. DBA/2J mice with wild type GPNMB develop very mild iris disease with no retinal ganglion cell loss [103]. The DBA/2J has immune system defects [104,105] and immune roles for GPNMB in glaucoma must be investigated further, as GPNMB can function as a feedback regulator of macrophage activation [95]. The upregulation of GPNMB in the primate model and in optic nerve head astrocytes from glaucomatous human donors suggests that proper immune function of astrocytes, as nonprofessional antigen presenting cells, may require GPNMB. Possible immune roles for GPNMB in optic nerve head astrocytes should be examined.

## Conclusion

We have identified a small group of genes differentially expressed in the optic nerve head of primates with experimental hypertensive glaucoma. Most have no previously characterized roles in glaucoma, yet many have previously identified roles elsewhere that suggest roles in glaucoma. Our most novel finding is that in mild glaucoma, before the appearance of visual field defects, retinal ganglion cell axons may make a regenerative attempt. The upregulation of neuroprotective genes in the glaucomatous optic nerve head supports this. Further investigations should determine whether neuroprotective molecules identified here can increase retinal ganglion cell survival in animal models of glaucoma.

## Methods

### Animals

Four cynomolgus macaques (*Macaca fascicularis*) had unilateral experimental ocular hypertension (4 experimental eyes and 4 control eyes), one macaque had unilateral "spontaneous" ocular hypertension, and 2 macaques had normal intraocular pressure and were used as controls. Data for the samples is presented in Table 1 and Table 2.

### Animal model of ocular hypertension

Unilateral laser scarification of the trabecular meshwork [37,38] was performed in 4 female cynomolgus macaques with a standard, clinical argon laser, or green diode laser and slit lamp delivery system. This was used to place 50 to 250 spots, 50 or 75 μm in diameter (1.0–1.5 W of energy; 0.5 to 0.8-second duration), over approximately 270° of the angle circumference while leaving one quadrant untreated. If increased intraocular pressure was not achieved, laser surgery was repeated at 3- to 4-week intervals, after ocular inflammation subsided. Anesthesia for laser surgery and fundus photography was induced with injections of intramuscular (i.m.) ketamine hydrochloride (10 mg/kg), supplemented with injections of i.m. medetomidine (15–75 μg/kg). A fifth macaque, sample 529, was used in two experiments that involved cannulating the anterior chamber, 1 and 2 years prior to developing elevated intraocular pressure. This macaque is referred to as the spontaneous glaucoma sample, as it is unclear as to whether either of these events were causes of the intraocular pressure elevation.

Under ketamine anesthesia, intraocular pressure was monitored with a minified Goldmann applanation tonometer [39] (Haag-Streit, Koniz, Switzerland), as well as a handheld applanation tonometer (Tono-pen XL; Mentor O & O, Norwell, Mass) if head or eye movements made accurate Goldmann readings impossible. The untreated, contralateral eye served as a normal control. Stereoscopic fundus photography was used to determine optic nerve cupping before and after laser treatment as well as before sacrifice.

For sacrifice, anesthesia was induced with i.m. ketamine (10 mg/kg), followed by deep pentobarbital anesthesia (15 mg/kg intravenous (i.v.)). All macaque work was performed under the guidelines of the ARVO Statement for the Use of Animals in Ophthalmic and Vision Research.

### GDx VCC scanning laser polarimetry

Monkeys were anesthetized with a combination of i.m. ketamine and medetomidine, followed by isoflurane, an inhalation anesthetic agent. Monkeys were maintained at, or near, surgical anesthetic depth for the scanning procedure using the GDx VCC (Zeiss, Dublin, CA). A 10 mm plano contact lens was applied to ensure adequate corneal hydration. Corneal birefringence compensation was calculated at baseline and verified by 3 or more compensated macular scans at each time point. During each session, 3 to 5 separate peripapillary scans were taken and the mean was calculated. The scan head was realigned between scans to generate discrete images. Definitions of the individual parameters and the technology of the instrument are described elsewhere [40-43].

### Tissue collection

Following sacrifice, the posterior segment of each eye was placed into RNALater (Ambion) and kept at 4°C until dissection was performed. The optic nerve head was carefully dissected from the eye and under the dissecting microscope, the sclera, meningeal sheaths and all traces of pigmented cells were removed. The resulting cylinder was then bisected with a sharp sterile blade and the border of myelinated nerve was visualized. A cut was made to include a sliver of approximately 1 mm of the myelinated nerve from the samples. Total RNA was isolated in TRIzol (Invitrogen Life Technologies, Carlsbad, CA), and tissue was minced in TRIzol with a scalpel blade. On average 1.5 μg of RNA was isolated per sample. After isolation, RNA was precipitated and resuspended in 10 μl nuclease-free water. RNA quality was verified by processing 250ng of RNA by capillary electrophoresis. RNA quantity and purity was estimated by measuring absorbance at 260 nm and absorbance ratios at 260/280 nm.

### Evaluation of nerve damage

To evaluate optic nerve damage, cross sections were taken from myelinated optic nerves fixed in 4% paraformaldehyde, osmicated, embedded in plastic and stained with p-phenylenediamine (PDA). After PDA, degenerated axons stain in dark brownish-red color whereas normal axons appear as black circles with a clear center due to staining of myelin. Digital images were taken at 2× magnification, so that the entire cross-sectional area of the nerve was within the lens field. Images were imported into Optimas software (Bothel, WA), for measurement of total circumference area and areas with axonal degeneration. The area occupied by degenerated axons was measured and expressed as the percentage of the total cross-sectional area. "Mild" axon loss was defined as a loss of up to a third (33%) of myelinated axon area, "moderate" axon loss when there was loss up to two thirds (66%) of myelinated axon area, and "advanced" axon loss was when the loss in axon area surpassed 66% of the total myelinated area as in [44].

### Reverse Transcription and Quantitative Real Time PCR

qRT-PCR was performed using 1 μg of total RNA isolated from the corresponding macaque optic nerve head. Reverse transcription, primer design and quantitative real time PCR were described previously in detail [15]. Primer sequences are provided [see Additional file 4]. 18S RNA or RPL19 was used as endogenous control. Fold change (i.e. fold increase or fold decrease) was calculated within each individual macaque between paired control and experimental eyes.

### Confirmation of selected genes in optic nerve head astrocytes from Caucasian American glaucomatous and control donors

Cytoplasmic RNA was isolated from cultured optic nerve head astrocytes (passage 3) from nine different glaucoma donors and nine different normal donors. The characteristics of the glaucoma donors are provided [see Additional file 7 and Additional file 8]. RNA from cultured optic nerve head astrocytes belonging to one eye of each of the donors was used. For quantitative real time PCR (qRT-PCR) conditions and primer sequences see Supplemental Table 2. For each cell culture, PCR reactions were performed in triplicate. Amplification of 18S ribosomal RNA was performed in all samples to normalize gene expression values. The threshold cycle (Ct) values of each sample and the standards were loaded into Microsoft Excel for further analysis. The mean and SEM of relative expression level were calculated for all target genes. Gene expression levels in all samples were expressed as the ratio of the gene of interest to 18S expression. Statistical analysis of the results was done using the statistical functions of the Graphpad Prism 4.0 software. Significant differences between the means were set at p < 0.05 (ANOVA followed by Bonferroni correction). For each target and the corresponding control gene, we used six individual normal and six glaucomatous donors simultaneously, each donor in triplicate. This was done to run all samples plus standards simultaneously in one plate.

### Monkey tissues for immunohistochemistry

Ocular tissue from 1 female cynomolgus monkey (aged 5 years) and 3 female and 2 male rhesus monkeys (*Macaca mulatta*) (aged 3–5 years), with monocular experimental glaucoma, were used to detect selected protein gene products in the optic nerve head and retina [see Additional file 5 and Additional file 6] Surgical procedure and tissue processing were described earlier in detail [45]. The monkeys were perfused through the heart with 4% paraformaldehyde in 0.1 M phosphate-buffered saline (PBS) pH 7.4, following deep pentobarbital anesthesia. After enucleation, the eyes were immersed in 4% paraformaldehyde for 12–24 h and the optic nerve head (with 2–3 mm of myelinated nerve) was dissected from surrounding tissues. Tissue was extensively washed in 0.2% glycine in PBS and processed for paraffin embedding. The retina and optic nerve were used for immunohistochemistry.

### Immunohistochemistry

Immunocytochemistry was performed on 6 μm sagittal sections of the optic nerve head containing retina and myelinated nerve. The specificity and optimal dilution of the antibodies were tested using monkey optic nerve head tissues. We used rabbit polyclonal antibodies against stathmin (1:50; Cell Signaling Technology); growth-associated protein 43 (GAP43), a marker for axonal growth (1:200; Santa Cruz Biotechnology); and neurofilament heavy (NF200) (1:250, Sigma St. Louis, MO). The polyclonal anti-NF200 antibody recognizes both phosphorylated and dephosphorylated forms of the 200-kDa neurofilament. We also used a monoclonal antibody against phosphorylated neurofilament heavy (1:250; SMI31, Sternberger Monoclonals, Baltimore, MD) that reacts only with a phosphorylated epitope in extensively phosphorylated forms of this polypeptide. We used monoclonal antibodies against GPNMB (1:500; Abnova Corporation, Taiwan) and protein disulfide isomerase (PDI), a marker of endoplasmic reticulum vesicles (1:200; Stressgen Biotechnologies, Canada). To localize selected gene products to optic nerve and retinal astrocytes, we performed double immunohistochemistry using mono- and polyclonal antibodies against glial fibrillary acidic protein (GFAP) (1:400 and 1:200 respectively; Sigma), an astrocyte marker. To localize stathmin to oligodendrocytes, we used a mouse monoclonal antibody against Adenomatus polyposis coli (APC), a marker for oligodendroglial cells (1:200; Calbiochem). We used secondary antibodies Alexa 488 and Alexa 568 (1:400; Molecular Probes, Eugene, OR). Slides were mounted with Prolong^® ^mounting media containing DAPI for nuclear staining. For negative controls, the primary antibody was replaced for the appropriate non-immune serum. To control for cross reactivity in double immunofluorescence, sections were incubated with primary antibody followed by the wrong species' secondary antibody. Sections of normal and glaucomatous eyes were stained simultaneously to control for variations in immunostaining. Slides were examined in a Nikon Eclipse 80*i *microscope (Tokyo, Japan) equipped with epifluorescent illumination and digital cameras (Photometrics). The images were processed using Meta Imaging Series software (Molecular Devices) and stored as computer files.

### Oligonucleotide microarray hybridization

For microarray hybridization, cDNA was synthesized from 50ng of total RNA using the Superscript Choice System (Life Technologies, Gaithersburg, MD) and T7-(dT) 24 primer (GENSET, La Jolla, CA) with an additional step of linear amplification. Amplicon length was on the order of 100 bp. *In vitro *transcription was done with purified double-stranded cDNA as the template in the presence of biotinylated UTP and CTP using a Bioassay High Yield RNA Transcript Labeling Kit (Enzo Diagnostics, Farmingdale, NY). Biotinylated cRNA was purified with Qiagen RNAeasy cleanup (Qiagen, Valencia, CA).

Hybridization of labeled cRNA to Affymetrix HG-U133A (cynomolgus macaque 529) and HG-U133Av2 (all other cynomolgus macaques) chips was done using the GeneChip Instrument System (Affymetrix, Santa Clara, CA) at the GeneChip Core Facility of Washington University. Arrays were washed and stained with strepavidin-phycoerythrin (Molecular Probes, Eugene, OR). Arrays were scanned with the Agilent GeneArray Scanner (Agilent Technologies, Palo Alto, CA). The output Affymetrix .CEL files were generated and scaled to target intensity 1,500 with the Affymetrix Microarray Suite software package (version 5.0). The details of each GeneChip used for the primate model are provided [see Additional file 9].

### Bioinformatic and statistical analysis of oligonucleotide microarray data

All analyses of microarray .CEL files were done using the open source statistical package R version 2.7.0 [45] and Bioconductor release 2.1 [46]. The complete R script containing our analyses for the control and experimental glaucoma (ExpG) macaques has been provided [see Additional File 10], which allows the reader to perform the identical analysis using provided raw data from the current study [see Additional Files 11, 12, 13, 14, 15, 16, 17].

### Oligonucleotide microarray normalization

Prior to any low-level or high-level analysis, probe sets on all chips were first remapped to updated UniGene probe set definitions (version 6) intended for *Homo sapiens *and *Pan troglodytes *(chimpanzee) downloaded from [46]. After remapping probe sets to the latest UniGene cluster IDs, the 'combineAffyBatch' function, from the Bioconductor 'matchprobes' library [47] version 1.8.1, was used to merge Affymetrix HG-U133A and HG-U133Av2 chips prior to normalization. All chips were normalized in one batch with the robust multiarray normalization (RMA) algorithm [48] using the 'rma' function, from the Bioconductor 'affy' library version 1.17.3 [49], with default settings. Our empirical observations and those of others working in nonhuman primates [50] determined RMA normalization to be the most accurate of several tested. Each biological sample was then scaled to mean zero, variance one using the 'normalize' function in the R 'som' library version 0.3–4 to minimize technical artifacts prior to statistical testing. Details of the parameters of these methods are presented in the R script provided [see Additional File 10].

### Statistical testing of control versus glaucoma groups

R statistical language implementations of Rank Products (RP) [51] ('RankProd' library version 2.8.0) and Significance Analysis of Microarrays (SAM) [52] ('siggenes' library version 1.12.0) were downloaded from the Bioconductor website . A significance cutoff of p = 0.05 was required to be deemed significantly differentially expressed. Each individual eye was used as an independent biological sample. RP and SAM produce q-values which are simultaneously adjusted for false discovery rate [53,54]. Details of the parameters of these methods are presented in the R script provided [see Additional File 10].

We performed two rounds of statistical testing and ad-hoc filtering. One was performed to identify genes that changed across all stages of ExpG, and the next was to preferentially identify genes that changed in the mild stages of ExpG. Specifically, we tested for genes that changed significantly between all eight control eyes and all five ExpG eyes (Table 3), and also tested for genes that changed significantly between all eight control eyes and the three mild ExpG eyes (577, 578, 579; Table 4). The additional ad-hoc filtering for each round was performed as described below.

### Calculation of fold change

Fold change (i.e. fold increase or fold decrease) was calculated between experimental and control groups by averaging technical replicates together for each biological sample, averaging all control biological and all experimental biological samples, and dividing the experimental mean by the control mean for upregulated probe sets, or dividing the control mean by the experimental mean and appending a negative sign for downregulated probe sets.

Standard error of fold change was calculated for group comparisons using the expression values for each biological sample. For individual, paired biological sample calculations, standard error of fold change was calculated using technical replicate values for the corresponding biological sample.

### Ad-hoc filtering of results of statistical testing by absent/present calls and fold change for all experimental glaucoma eyes versus all control sample eyes

In addition to selection by p-value, we applied an arbitrary fold change (i.e. fold increase or fold decrease) cutoff of 45% between control and ExpG groups. This step was taken to ensure that truly differentially expressed transcripts were identified; others have noted that larger fold changes (also by Affymetrix microarray) correlate more closely with qRT-PCR measurements than smaller changes [55].

The next filter we applied was by Affymetrix detection call. Probe sets called 'present' (p <= 0.05 by Wilcoxon one-sided signed rank test; [56,57]) with the 'mas5calls' function from the 'affy' library in approximately half of the total number of GeneChips (16 out of 33 total chips) were retained for further analysis. We chose this relatively stringent arbitrary cutoff to ensure that only reliably detected transcripts would be retained for further investigation.

Finally, we applied an additional fold change filter. We stipulated that probesets retained for the final list of significant differentially expressed genes must also pass a fold change cutoff of 45% in at least two of the five paired, experimental samples. This final step was done to ensure that transcripts that had extreme changes in a single sample had not biased the calculated group fold changes.

### Ad-hoc filtering of results of statistical testing by absent/present calls and fold change for three mild experimental glaucoma eyes versus all control sample eyes

This analysis was performed in the same manner as for the entire group comparison above, using only the three mild ExpG samples (577, 578, 579) and excluding the two advanced ExpG samples (566, 529). As for the entire group comparison above, all eight control samples were used. Following statistical testing, probe sets that were called 'present' in 14 out of 28 chips, changed 45% between control and mild ExpG groups, and also changed 45% between control and ExpG eyes in at least two of the three paired mild samples (577, 578, 579) were identified as significantly differentially expressed between groups.

### Prediction of cell types expressing differentially expressed transcripts

Two previously published *in vitro *datasets of immunopanned cultures of pure optic nerve head astrocytes were used for calculation of 'present' probe sets [11] (NCBI GEO GSE2378); [23] (NCBI GEO GSE758). These datasets were remapped to human UniGene-based definitions (Version 6) [46] and RMA normalized [48] as described for the macaque data. Probe sets were considered expressed by optic nerve head astrocytes if called 'present' in at least 25% of all microarray chips from either [23] or [11]. These were matched to any significant genes from the present study on multiple identifiers.

Retinal ganglion cell expression data from EST libraries and microarray was obtained, respectively, from [58] and [59], and matched on multiple identifiers. Probe sets were considered expressed by retinal ganglion cells if detected in retinal ganglion cells in either of the two studies.

Additional information from the scientific literature was used to predict cell-type expression and to exclude any false positives resulting from retinal contamination during the dissection.

## Abbreviations

ECM: Extracellular (matrix); (ExpG): experimental glaucoma; (GEO): Gene Expression Omnibus; (GFAP): glial fibrillary acidic protein; (LC): lamina cribrosa; (ONH): optic nerve head; (POAG): primary openangle glaucoma; (PCR): polymerase chain reaction; (qRT-PCR): quantitative real-time PCR; (RP): Rank Products; (RNFL): retinalnerve fiber layer; (SAM): Significance Analysis of Microarrays; (VCC): variable corneal compensation.

## Authors' contributions

All animal work was performed by PLK and CAR at the Department of Ophthalmology & Visual Sciences, University of Wisconsin, Madison. CAR and MRH wrote sections of the manuscript. MRH designed the study, made figures, and performed microscopy. OAO performed dissections and RNA isolation. OAO, KSK, and WL performed qRT-PCR experiments. KSK performed analysis of array data and wrote the manuscript. All authors critically read and revised the manuscript.

## Supplementary Material

Additional file 1 **kompass_et_al_BMC_Neuroscience.** Fundus photography images for selected ExpG samples. Click here for file

Additional file 2 **kompass_et_al_BMC_Neuroscience.** GDx VCC scans for selected ExpG samples. Click here for file

Additional file 3 **Kompass_et_al_BMC_Neuroscience.** Individual stages of microarray data processing that generated the results shown in **Table 3 **and **Table 4**. Click here for file

Additional file 4 **kompass_et_al_BMC_Neuroscience.** Primer sequences for quantitative real-time PCR. Click here for file

Additional file 5 **kompass_et_al_BMC_Neuroscience.** Clinical information for macaque samples used for immunohistochemistry. Click here for file

Additional file 6 kompass_et_al_BMC_Neuroscience. Intraocular pressure measurements, for six paired ExpG samples used for immunohistochemistry, from day of first laser in ExpG eye to sacrifice. Day 0 (vertical line) is the first time intraocular pressure was above 25 mm Hg. Click here for file

Additional file 7 **kompass_et_al_BMC_Neuroscience.** Clinical information for control Caucasian American donors used to generate primary cultures of ONH astrocytes. Click here for file

Additional file 8 **kompass_et_al_BMC_Neuroscience.** Clinical information for POAG Caucasian American donors used to generate primary cultures of ONH astrocytes. Click here for file

Additional file 9 **kompass_et_al_BMC_Neuroscience.** Details of Affymetrix chips used in the primate study. Click here for file

Additional file 10 **kompass_et_al_BMC_Neuroscience.** R statistical language script containing microarray analysis for **Table 3 **and **Table 4**. Includes instructions for how to install required R and Bioconductor libraries to repeat the analysis. Script may be pasted into an R session or called using the 'source' function in R. Click here for file

Additional file 11 **kompass_et_al_BMC_Neuroscience.** Raw microarray data for sample 577. Click here for file

Additional file 12 **kompass_et_al_BMC_Neuroscience.** Raw microarray data for sample 578. Click here for file

Additional file 13 **kompass_et_al_BMC_Neuroscience.** Raw microarray data for sample 579. Click here for file

Additional file 14 **kompass_et_al_BMC_Neuroscience.** Raw microarray data for sample 566. Click here for file

Additional file 15 **kompass_et_al_BMC_Neuroscience.** Raw microarray data for sample 529. Click here for file

Additional file 16 **kompass_et_al_BMC_Neuroscience.** Raw microarray data for sample k605. Click here for file

Additional file 17 **kompass_et_al_BMC_Neuroscience.** Raw microarray data for sample m590os. Click here for file
