# Supplementary material for: Bioinformatic and statistical analysis of the optic nerve head in a primate model of ocular hypertension
Source: BMC Neurosci. 2008 Sep 26;9:93. doi: 10.1186/1471-2202-9-93 (PMC2567987; doi:10.1186/1471-2202-9-93)
Supplement: Additional file 1 — kompass_et_al_BMC_Neuroscience. Fundus photography images for selected ExpG samples. [file 1471-2202-9-93-S1.doc]

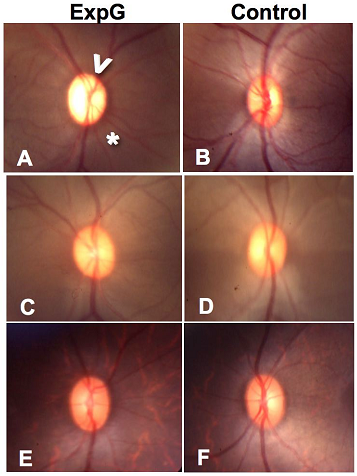


**Additional file 1.**

**Fundus photography from selected ExpG samples.**

Fundus photographs were taken on day of sacrifice. Fundus photography of experimental monkey 566 (**A,B;** 74% axon loss); 578 (**C, D;** 25% axon loss); 577 (**E,F;** 21% axon loss).

**(A, B)** Monkey 566 at 43 days after unilateral intraocular pressure elevation (Mean intraocular pressure experimental glaucoma = 31mm Hg; control = 16mm Hg). Monkey 566 (74% axon loss by count; **Table 2**) has an enlarged cup with undetermined margins at the edge of the disc (white arrowhead). The white asterisk marks a loss of reflectance in the retinal nerve fiber layer indicating a loss of retinal ganglion cells. **(C, D)** Monkey 578 at 234 days after unilateral intraocular pressure elevation (Mean intraocular pressure experimental glaucoma = 26mm Hg; control = 17mm Hg). **(E, F)** Monkey 577 at 76 days after unilateral intraocular pressure elevation (Mean intraocular pressure experimental glaucoma = 35mm Hg; control = 16mm Hg). There is little difference between the control and experimental glaucoma eyes in sample 577 (21% axon loss by count; **Table 2**) and sample 578 (25% axon loss by count; **Table 2**).
