# Supplementary material for: Bioinformatic and statistical analysis of the optic nerve head in a primate model of ocular hypertension
Source: BMC Neurosci. 2008 Sep 26;9:93. doi: 10.1186/1471-2202-9-93 (PMC2567987; doi:10.1186/1471-2202-9-93)
Supplement: Additional file 2 — kompass_et_al_BMC_Neuroscience. GDx VCC scans for selected ExpG samples. [file 1471-2202-9-93-S2.doc]

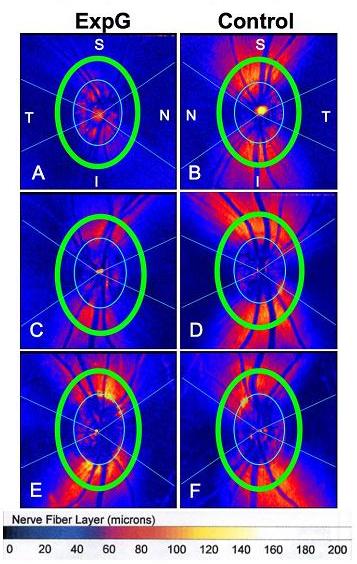


**Additional file 2.**

**GDx VCC scans from selected ExpG samples.**

Scans were performed on day of sacrifice. GDx VCC scans of monkey 566 (**A,B;** 74% axon loss); 578 (**C, D;** 25% axon loss); 577 (**E,F;** 21% axon loss).

The thickness of the RNFL is color-coded in the scans as in the bar below them. S, I, N, T in the figures (white) indicate orientation of the eye; **S**=superior, **I**=inferior, **N**=nasal, **T**=temporal. Scans were performed prior to sacrifice. **(A, B)** Monkey 566 at 43 days after unilateral intraocular pressure elevation (Mean intraocular pressure experimental glaucoma = 31mm Hg; control = 16mm Hg). The superior and inferior regions of monkey 566 (74% axon loss by count; **Table 2**) feature a very thinned RNFL. **(C, D)** Monkey 578 at 234 days after unilateral intraocular pressure elevation (Mean intraocular pressure experimental glaucoma = 26mm Hg; control = 17mm Hg). **(E, F)** Monkey 577 at 76 days after unilateral intraocular pressure elevation (Mean intraocular pressure experimental glaucoma = 35mm Hg; control = 16mm Hg). Very mild damage was observed in sample 577 (21% axon loss by count; **Table 2**) and in 578 (25% axon loss by count; **Table 2**).
