# Supplementary material for: Bioinformatic and statistical analysis of the optic nerve head in a primate model of ocular hypertension
Source: BMC Neurosci. 2008 Sep 26;9:93. doi: 10.1186/1471-2202-9-93 (PMC2567987; doi:10.1186/1471-2202-9-93)
Supplement: Additional file 4 — kompass_et_al_BMC_Neuroscience. Primer sequences for quantitative real-time PCR. [file 1471-2202-9-93-S4.doc]

**Additional file 4.**

**Primer sequences for quantitative real-time PCR.**

| **Gene** | **Forward primer (5'-3')** | **Reverse primer (5'-3')** |
| --- | --- | --- |
| APOE | CCGCCTCAAGAGCTGGTTC | CCACGGCAGCCTGCAC |
| BMP2 | CCAAACACAAACAGCGGAAAC | GGGAGCCACAATCCAGTCATT |
| CAPG^1^ | TGGACCTGGGCCAGAACA | TGCCCTGTCGCTCACTGTC |
| CAPG^2^ | AGAAGAGGTTTCCCATCTGCACCT | ATAGATCTTGCCACAGAGCCCGTT |
| GAP43 | GCTGAAGAGAACATAGAAGCTGTAGATG | CTTGGTCAGCCTCAGGTTCCT |
| GFAP | GAGTGGTACCGCTCCAAGTTTG | TGGCGCCGGTAGTCGTT |
| GPNMB | TCCGTGAGAATTCAGCATGG | AGCACATCATGAAATCGTTTGG |
| GPX1 | TGCCAGCTCCCAGCGA | CAAAGTTCCAGG CAACAT CGTT |
| NEFH | CCGAAT GCCACACGTAAACA | CGGTCATCTGTCAGTTGCACA |
| NEFL | ATGCAGAGTATCTGTTTGCTTGCA | TCACATTGCCGTAGATCCTGAAC |
| RBP1^2^ | GCCAACTTGCTGAAGCCAGACAAA | TTGCGGTCATCTATGCCTGTCAGA |
| RPL19 | GCCTGTGACGGTCCATTCC | TGGCTGTACCCTTCCGCTTA |
| STMN2 | CCCACGAACTTTAGCTTCTCCA | CTGGGCCTCCTGAGACTTTCTT |
| TGFβI | TATCAACGGGAAGGCGATCA | GTGTCTTGGCTGAGTCTGGGAT |
| TIMP1 | ACCAGAAGTCAACCAGACCACCTT | AAACACTGTGCATTCCTCACAGCC |

^1^Only used for qRT-PCR with cynomolgus macaques.

^2^Only used for qRT-PCR with human donors.
