# Supplementary material for: Bioinformatic and statistical analysis of the optic nerve head in a primate model of ocular hypertension
Source: BMC Neurosci. 2008 Sep 26;9:93. doi: 10.1186/1471-2202-9-93 (PMC2567987; doi:10.1186/1471-2202-9-93)
Supplement: Additional file 5 — kompass_et_al_BMC_Neuroscience. Clinical information for macaque samples used for immunohistochemistry. [file 1471-2202-9-93-S5.doc]

**Additional file 5.**

Clinical information for additional macaque samples used for immunohistochemistry.

| **Macaque nº/sex/age (yr)** | **Evaluation** | **Duration (weeks / days)*** | **IOP (mmHg)** | **C/D ratio** | **Axonal loss (%)** | **Species** |
| --- | --- | --- | --- | --- | --- | --- |
| 1/M/3 | Mild | 22 / 153 | ExpG: 25.2 ± 11.2 | ExpG: 0.9 | 25 | Rhesus |
|  |  |  | C: 15.2 ± 2.2 | C: 0.3 |  |  |
| 2/M/3 | Mild | 23 / 168 | ExpG: 19.7 ± 2.2 | ExpG: 0.2 | 28 | Rhesus |
|  |  |  | C: 13.9 ± 1.9 | C: 0.1 |  |  |
| 3/F/5 | Mild | 23 / 160 | ExpG: 30.2 ± 11.7 | ExpG: 0.2 | 15 | Rhesus |
|  |  |  | C: 17.0 ± 1.3 | C: 0.2 |  |  |
| 4/F/5 | Moderate | 38 / 264 | ExpG: 29.4 ± 13.0 | ExpG: 0.7 | 58 | Rhesus |
|  |  |  | C: 18.9 ± 2.9 | C: 0.2 |  |  |
| 5/F/5 | Moderate | 45 / 317 | ExpG: 22.2 ± 2.5 | ExpG: 0.9 | 37 | Cynomolgus |
|  |  |  | ExpG: 17.0 ± 1.7 | ExpG: 0.2 |  |  |
| 6/F/8 | Mild | 19 / 132 | ExpG: 26.5 ± 4.3 | ExpG: 0.2 | 20 | Rhesus |
|  |  |  | ExpG: 15.9 ± 2.4 | ExpG: 0.1 |  |  |

ExpG: experimental glaucoma eye. C: contralateral control eye. IOP measurements represent mean ± SD. Samples 5 and 6 were lasered to produce ExpG in both eyes. * Length of time between initial IOP elevation and sacrifice.
