# Supplementary figures and images for: Bioinformatic and statistical analysis of the optic nerve head in a primate model of ocular hypertension
Source: BMC Neurosci. 2008 Sep 26;9:93. doi: 10.1186/1471-2202-9-93 (PMC2567987; doi:10.1186/1471-2202-9-93)

IOP (mmHg)

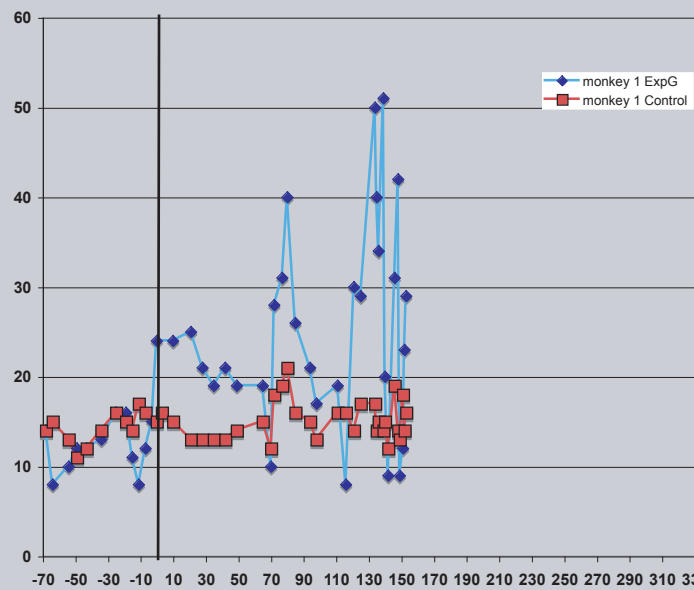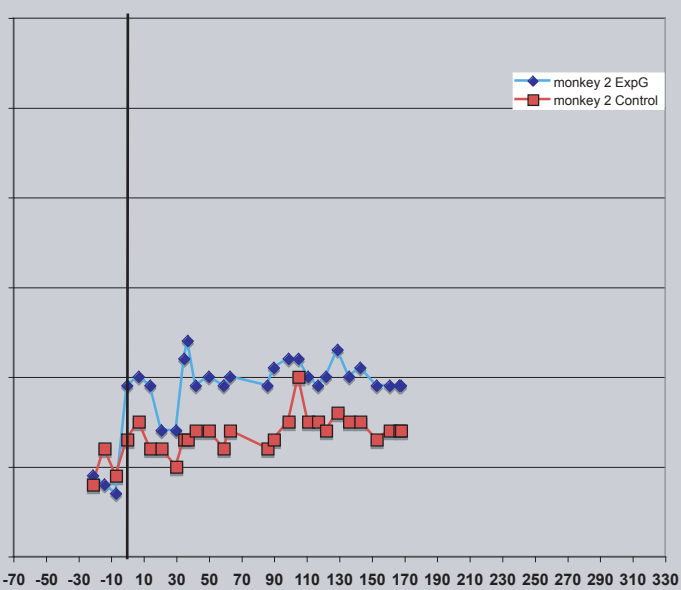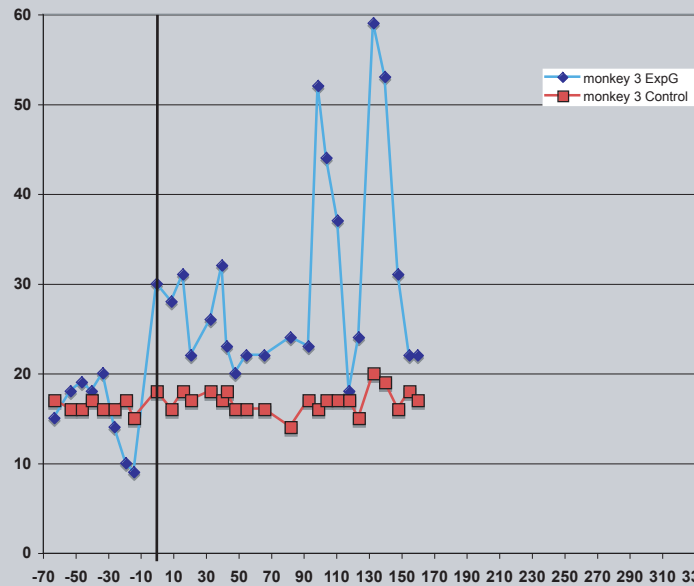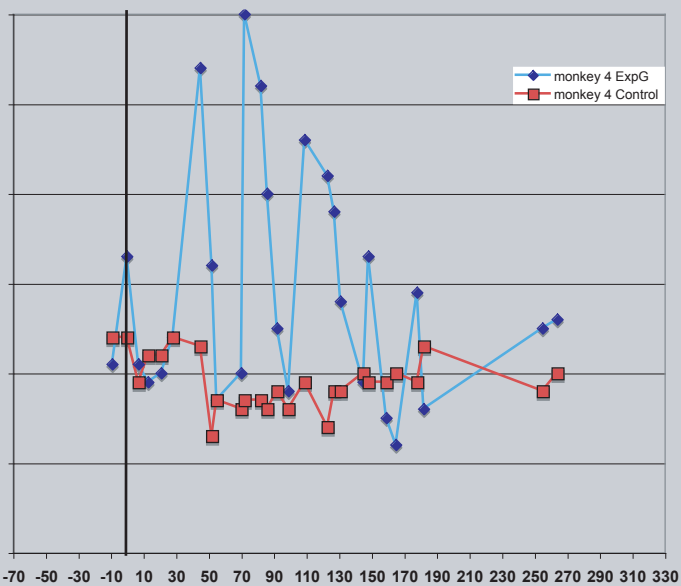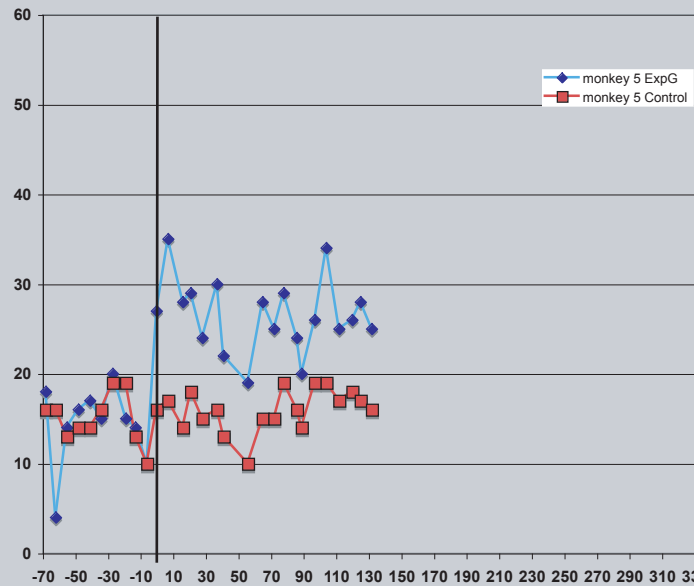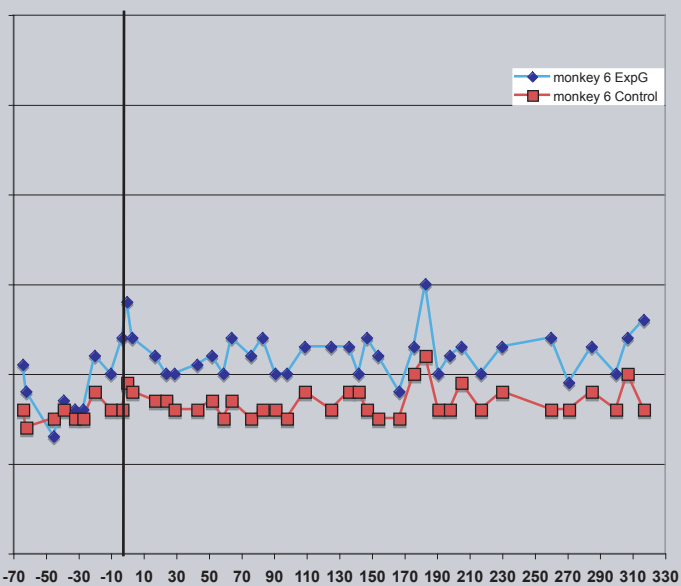

Days of Elevated IOP

Supplement: Additional file 6 — kompass_et_al_BMC_Neuroscience. Intraocular pressure measurements, for six paired ExpG samples used for immunohistochemistry, from day of first laser in ExpG eye to sacrifice. Day 0 (vertical line) is the first time intraocular pressure was above 25 mm Hg. [file 1471-2202-9-93-S6.pdf]
