# Supplementary material for: Bioinformatic and statistical analysis of the optic nerve head in a primate model of ocular hypertension
Source: BMC Neurosci. 2008 Sep 26;9:93. doi: 10.1186/1471-2202-9-93 (PMC2567987; doi:10.1186/1471-2202-9-93)
Supplement: Additional file 7 — kompass_et_al_BMC_Neuroscience. Clinical information for control Caucasian American donors used to generate primary cultures of ONH astrocytes. [file 1471-2202-9-93-S7.doc]

**Additional file 7.**

**Clinical information for control Caucasian American donors used to generate primary cultures of ONH astrocytes.**

| **Donor ID** | **Sex** | **Age** | **Cause of death** | **Time of death** | **Time of enucleation** |
| --- | --- | --- | --- | --- | --- |
| CA1 | F | 46 | Myocardial infarction | 0:37 | 5:45 |
| CA2 | M | 52 | Congestive heart failure | 6:25 | 9:40 |
| CA3 | F | 68 | Sepsis | 16:20 | 20:30 |
| CA4 | F | 72 | Myocardial infarction | 20:50 | 1:52 |
| CA5 | M | 49 | Metastatic esophageal cancer | 4:25 | 8:35 |
| CA6 | M | 50 | Aspiration pneumonia | 13:56 | 18:00 |
| CA7 | M | 65 | Cardiac arrest | 1:01 | 5:15 |
| CA8 | M | 42 | Hypotension, cancer | 6:21 | 9:30 |
| CA9 | M | 57 | Cardiac arrest | 3:57 | 9:55 |

CA: Caucasian American. M = male; F = female.
