# Supplementary material for: Bioinformatic and statistical analysis of the optic nerve head in a primate model of ocular hypertension
Source: BMC Neurosci. 2008 Sep 26;9:93. doi: 10.1186/1471-2202-9-93 (PMC2567987; doi:10.1186/1471-2202-9-93)
Supplement: Additional file 8 — kompass_et_al_BMC_Neuroscience. Clinical information for POAG Caucasian American donors used to generate primary cultures of ONH astrocytes. [file 1471-2202-9-93-S8.doc]

**Additional file 8.**

Clinical information for Caucasian American donors with POAG used to generate primary cultures of ONH astrocytes.

| **Donor ID** | **Age** | **Sex** | **Cause of death** | **Time of death** | **Time of enucleation** | **Severity of glaucoma *** | **Glaucoma treatment** |
| --- | --- | --- | --- | --- | --- | --- | --- |
| **00-6 R** | 56 | F | Spontaneous aneurysm hemorrhage | 05:21 | 09:00 | Mild (NA) | Latanoprost |
| **05-12 R** | 77 | F | Pancreatic cancer | 21:22 | 23:55 | Mild (22%) | Latanoprost, brimonidine |
| **02-1 R** | 69 | M | Acute respiratory failure | 06:59 | 11:00 | Moderate (58%) | NA |
| **04-1 L** | 79 | F | Chronic heart failure | 21:15 | 07:35 | Mild (25%) | NA |
| **04-5 L** | 72 | F | Chronic heart failure, renal failure | 05:22 | 10:35 | Moderate (55%) | NA |
| **02-7 L** | 71 | M | Cerebral-vascular accident | 23:50 | 03:55 | Moderate (45%) | NA |
| **05-2 R** | 70 | M | Cardiac arrest | 01:18 | 04:15 | Mild (20%) | Latanaprost |
| **02-10 R** | 80 | F | Acute respiratory failure | 06:00 | 12:00 | Advanced (85%) | NA |
| **04-16 R** | 80 | M | Chronic heart failure | 23:30 | 02:30 | Advanced (78%) | Timolol- dorzolamide, brimonidine |

*Severity of glaucoma was based on evaluation of the myelinated optic nerve and clinical ophthalmic history, and was based on percent axonal loss cutoffs as for monkey samples (see Methods ‘Evaluation of nerve damage’ section). When available, remaining axon percentages are shown in parentheses.

NA: Data not available. M = male; F = female.
